# Tipping the Scales With Zebrafish to Understand Adaptive Tumor Immunity

**DOI:** 10.3389/fcell.2021.660969

**Published:** 2021-05-20

**Authors:** Kelly Z. Miao, Grace Y. Kim, Grace K. Meara, Xiaodan Qin, Hui Feng

**Affiliations:** ^1^Department of Pharmacology & Experimental Therapeutics, Boston University School of Medicine, Boston, MA, United States; ^2^Department of Medicine, Section of Hematology and Medical Oncology, Boston University School of Medicine, Boston, MA, United States

**Keywords:** cancer, immunotherapy, zebrafish, adaptive tumor immunity, TME, lymphocyte

## Abstract

The future of improved immunotherapy against cancer depends on an in-depth understanding of the dynamic interactions between the immune system and tumors. Over the past two decades, the zebrafish has served as a valuable model system to provide fresh insights into both the development of the immune system and the etiologies of many different cancers. This well-established foundation of knowledge combined with the imaging and genetic capacities of the zebrafish provides a new frontier in cancer immunology research. In this review, we provide an overview of the development of the zebrafish immune system along with a side-by-side comparison of its human counterpart. We then introduce components of the adaptive immune system with a focus on their roles in the tumor microenvironment (TME) of teleosts. In addition, we summarize zebrafish models developed for the study of cancer and adaptive immunity along with other available tools and technology afforded by this experimental system. Finally, we discuss some recent research conducted using the zebrafish to investigate adaptive immune cell-tumor interactions. Without a doubt, the zebrafish will arise as one of the driving forces to help expand the knowledge of tumor immunity and facilitate the development of improved anti-cancer immunotherapy in the foreseeable future.

## Introduction

Dr. Harold F. Dvorak wrote in 1986 that solid tumors are comprised of two discrete compartments, the malignant cells and the stroma in which they are dispersed, creating an environment that resembles a “wound that does not heal” (Dvorak, 1986). One challenge in cancer treatment–healing the “wound”–stems from the difficulty in fully understanding the mechanisms by which cancer cells escape immunosurveillance in the tumor microenvironment (TME). In recent years, immunotherapy has made revolutionary advances in our war against cancer. The successful development and application of a newer generation of cancer immunotherapy, such as immune checkpoint blockade and chimeric antigen receptor T cell (CAR-T), have led to improved outcomes in cancers including hematologic malignancies, melanoma, lymphomas, and lung cancers (Tang et al., 2017; Gong et al., 2018). Despite these progresses, immunologically “cold” tumors, encompassing a broad spectrum of solid tumors such as breast cancer, pancreatic cancer, neuroblastoma, and glioblastoma, present unique challenges for immunotherapy (Bonaventura et al., 2019). Some of the underlying causes are the lack of tumor antigens, a deficit in antigen-presenting B cells, and/or impaired trafficking of activated T cells into the TME (Bonaventura et al., 2019). For immunotherapies to become highly effective for cancers, the following challenges must be addressed: (1) enhancement of major histocompatibility complex (MHC) expression in tumor cells for sufficient presentation of tumor-associated antigens and (2) regulation of cytokines and manipulation of the TME to improve effector T-cell infiltration into “cold” tumors (Stambrook et al., 2017).

For decades, mice have represented the primary animal model and major contributor to cancer research including tumor immunity research (Callahan et al., 2014). However, this model has limitations. One major drawback is that drugs’ effectiveness and safety evaluated by pre-clinical murine models often cannot be reproduced in clinical trials (Rossa and D’Silva, 2019). This issue calls for the inclusion of additional animal models to obtain preclinical data that can be replicated in multiple systems in order to boost the success rates of clinical testing. Another drawback of the murine model is the difference in telomerase between human and mouse cells (Cheon and Orsulic, 2011). Specifically, most mouse cells have active telomerase throughout adulthood while human adult cells have largely inactive telomerase (Cheon and Orsulic, 2011). Interestingly, the zebrafish possesses human-like telomeres, which gradually decline with age, allowing its use to replicate similar genomic instabilities seen in humans (Carneiro et al., 2016). Another unique advantage of the zebrafish is that they are raised in non-sterile conditions, making them physiologically relevant to study immune responses (Iwanami et al., 2017). Coupled with their imaging capacities, the zebrafish enables real-time and non-invasive monitoring of tumor-immune cell interactions through differential fluorescent labeling of cells.

Through exhaustive comparison of the human and zebrafish genome assemblies, it was found that 82% of disease-causing human genes have at least one zebrafish ortholog (Howe et al., 2013). This level of conservation has led to the development of a wide array of cancer models in zebrafish (Casey and Stewart, 2018; Hason and Bartůněk, 2019; Casey and Stewart, 2020; Elliot, 2020). Through the years of ongoing research, the pathological similarities of cancer and preservation of the immune components have also been well established between zebrafish and humans. For instance, many oncogenic pathways are conserved in zebrafish along with similar development processes in hematopoiesis and the immune system (Traver et al., 2003; Davidson and Zon, 2004; White et al., 2013; Gore et al., 2018; Casey and Stewart, 2020). Hence, the zebrafish represents a physiologically relevant model system for tumor immunity research. Here, we summarize the suitability and unique advantages of the zebrafish in investigating adaptive tumor immunity. We also provide a few examples of early studies to demonstrate its feasibility in expanding the knowledge of this field and its great potentials in advancing cancer immunotherapy.

## In the Same Sea: High Conservation of the Immune System

**FIGURE 1:**
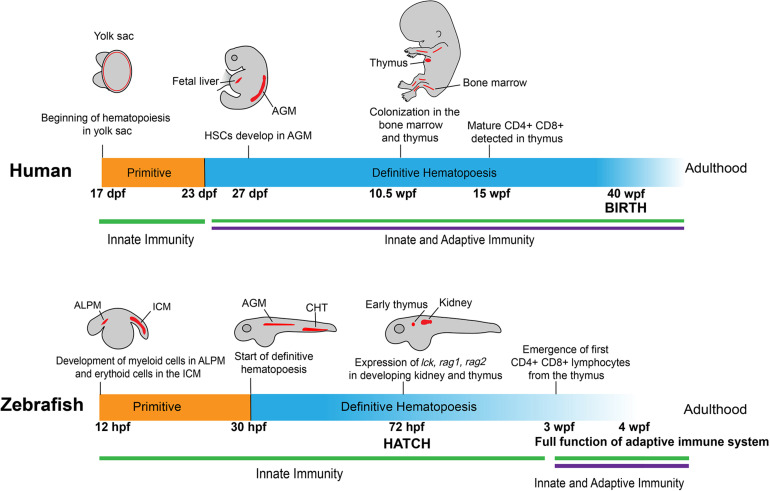
Development of human and zebrafish immune systems. The development of the immune system starts with hematopoiesis at ∼17 dpf in humans and 12 hpf in the zebrafish, with myeloid and erythroid cells arising in the ALPM and ICM, respectively (Jagannathan-Bogdan and Zon, 2013). In humans, myeloerythroid progenitor cells seed in the yolk sac before HSCs appear in the AGM at 27 dpf (Julien et al., 2016), a stage mirrored in zebrafish with the start of definitive hematopoiesis at 30 hpf in the AGM and transition into the caudal hematopoietic tissue (Jagannathan-Bogdan and Zon, 2013). At 72 hpf, vital markers for early lymphoid progenitors are present in developing immune organs, such as the early thymus and kidney in zebrafish (Willett et al., 1999; Langenau et al., 2004; Trede et al., 2004). This corresponds roughly to the colonization of immune cells in the bone marrow and thymus in the human fetus at 10.5 wpf (Kissa et al., 2008). Notably, at 72 hpf the zebrafish emerges from the chorion and into contact with the outside environment without fully developed CD4 + /CD8 + lymphocytes, which appear later at 3 wpf (Lam et al., 2004). This is contrasted to humans, in which lymphocytes are detectable at 12–13 wpf, well before birth at 40 wpf (Tavian et al., 2010). ALPM: anterior lateral plate mesoderm; ICM: intermediate cell mass; HSC: Hematopoietic stem cells; AGM: aorta-gonad-mesonephros.

The suitability of using the zebrafish to study tumor immunity stems from the fact that hematopoiesis and most immune components are highly conserved between humans and zebrafish, beginning from the embryonic stage into adulthood (Figure 1). Similar to humans, the zebrafish immune system is divided into two main branches: innate and adaptive. Despite some differences in the locations and timing of immune cell development, many pathways in primitive and definitive hematopoiesis, lymphocyte differentiation, along with key markers that define specific groups of immune cells are shared between zebrafish and humans (Traver et al., 2003; Iwanami, 2014; Gore et al., 2018).

### Timing of Development

The development of the immune system begins with the emergence of erythroid and myeloid lineages (Figure 1). In humans, this occurs at the Carnegie Stage (CS) 7 and 9, which correspond to 16–18.5 days post-conception (Ivanovs et al., 2017). In zebrafish, primitive macrophages appear at around 12 hours post-fertilization (hpf) (Wattrus and Zon, 2018). Interestingly, these primitive macrophages from the yolk sac follow an expedited differentiation pathway, possessing the ability to engulf pathogens and patrol the entire organism (Herbomel et al., 2001; Novoa and Figueras, 2012). A portion of these primitive macrophages will become neutrophils by 33 hpf (Harvie and Huttenlocher, 2015). During this primitive wave of development, the zebrafish expresses multiple homologous genes as mammals, including *lmo2*, *gata1a*, *scl*, and *cul4a* in the erythroid lineage and *pu.1* in the myeloid lineage (Yamada et al., 2001; Dooley et al., 2005; Galloway et al., 2005; Zhu et al., 2005; Yang et al., 2019).

Lymphoid cell development follows immediately after the appearance of erythroid and myeloid cells, similar to what has been observed at CS11 or 24 days post-conception in humans (Herbomel et al., 1999; Tavian et al., 2010; Carroll and North, 2014; Ivanovs et al., 2017). Markers of lymphoid progenitors, such as *rag1*, *rag2*, *lck*, and *ikaros*, present in the respective lymphoid progenitors and lymphocytes as those observed in humans, and are all detectable at the end of the primitive wave of hematopoiesis (Willett et al., 1997; Willett et al., 2001; Langenau et al., 2004; Jing and Zon, 2011). Although lymphoblast/lymphocyte markers arise as early as 3 days post fertilization (dpf), the zebrafish still rely on the innate immune system for defense against external threats. Starting at 4 dpf, four early hematopoietic markers, *c-myb*, *ikaros*, *runx2*, and *scl*, begin to express in the kidney of the zebrafish, marking the initiation of definitive hematopoiesis in this location (Murayama et al., 2006). Meanwhile, T cells develop in the thymus at a similar time and enter circulation at around 8 dpf (Page et al., 2013). Around 20 dpf, B progenitor cells develop in the dorsal aorta and posterior cardinal vein in zebrafish (Page et al., 2013). Adaptive immunity in the form of circulating lymphocytes does not fully mature until 3 weeks post-fertilization (wpf) (Willett et al., 1999; Trede et al., 2004; Novoa and Figueras, 2012). In humans, however, this lag does not exist. At 12–13 weeks, CD4 + and CD8 + T cells mature in the thymus of the developing human fetus and start circulating throughout the body before birth (Haynes et al., 1988; Jagannathan-Bogdan and Zon, 2013).

### Locations of Development

The locations in which the hematopoietic lineages develop between zebrafish and humans also differ somewhat (Figure 1). In humans, hematopoiesis begins in the yolk sac before colonization in the fetal liver and the production of hematopoietic stem cells (HSCs) in the aorta-gonad-mesonephros (AGM) region at around 27 dpf (Mikkola and Orkin, 2006; Ivanovs et al., 2017). The definitive wave of hematopoiesis that produces the adult immune system in humans occurs in the bone marrow (Wattrus and Zon, 2018). In zebrafish embryos, early hematopoiesis originates in the intermediate cell mass (ICM) for erythroid cells and the anterior lateral plate mesoderm (ALPM) for myeloid cells (Willett et al., 1999; Berman et al., 2005; Hogan et al., 2006; Jing and Zon, 2011). Similar to humans, there is a transitional period in which zebrafish hematopoiesis occurs in the AGM. Following this stage, the HSCs move to the caudal hematopoietic tissue, which functions comparably to the mammalian fetal liver. Finally, the cells move into the definitive hematopoietic organs, the thymus and kidney, from where the lymphoid cells begin to develop and later emerge (Langenau et al., 2004; Burns et al., 2005; Murayama et al., 2006). The definitive wave of hematopoiesis that produces the adult immune system occurs in the zebrafish kidney as opposed to the bone marrow in humans (Jagannathan-Bogdan and Zon, 2013; Wattrus and Zon, 2018).

### Conservation of Innate and Adaptive Immunity

Like humans, the zebrafish possess two main branches of immunity with fully fledged innate and adaptive components. In addition, the fish also possess two primary lymphoid organs, the kidney marrow (equivalent to the bone marrow in mammals) and thymus which shrinks in adult fish as in humans, as well as one secondary peripheral organ, the spleen in adult fish (Wattrus and Zon, 2018). However, one difference in terms of secondary immune organs is that the zebrafish, like other teleosts, lack lymph nodes. Instead, the vast majority of interactions between antigen-presenting cells (APCs) and lymphocytes occur in the spleen (Renshaw and Trede, 2012). Fish cells also express both MHC class I and II molecules, indicating the conserved interactions between the innate and adaptive immune systems (Fischer et al., 2013).

Zebrafish harbor the same fundamental system of innate immunity with leukocytes such as the entire macrophage lineage along with granulocytes like neutrophils and eosinophils (Traver et al., 2003; Novoa and Figueras, 2012). These cells mount a similar innate immune response as seen in mammals to local infections with the expression of characteristic cytokines such as TNF-α and IL-1β, which trigger the engulfment of pathogens by macrophages (Secombes et al., 2001; Traver et al., 2003). So far, 22 putative toll-like receptors (TLRs), including orthologs of all 10 human TLRs, have been discovered in zebrafish (Kanwal et al., 2014; Li et al., 2017).

In terms of adaptive immunity, as described above, zebrafish possess functional T and B cells after 3–6 weeks of development. T cell development occurs in the thymus of larval and adult fish, while mature T cells reside in the kidney marrow of adult fish. B cell development occurs in the pronephros of larval fish and kidney marrow of adult fish (Langenau et al., 2004; Trede et al., 2004). At 3 wpf, B cells become fully mature and active, in tandem with T cells as they emerge from the thymus (Lam et al., 2004). This indicates that the immune system in zebrafish reaches a mature stage at 3–6 wpf (Lam et al., 2004). However, one key difference exists between zebrafish and human B lymphocytes: three main classes of immunoglobulins have been discovered in zebrafish (IgD, IgM, and IgZ) versus five classes in humans (IgA, IgD, IgE, IgG, and IgM) (Zimmerman et al., 2011). Nevertheless, the overall similarities in the development of zebrafish and human immune systems allow for experimental modeling of human TME in zebrafish to visualize and understand the complex interactions between immune and tumor cells.

## Swimming Together: Fish Players in Adaptive Immunity

The status of adaptive immunity activation in the TME is a key predictor of prognosis for solid tumors (Bonaventura et al., 2019). Recent research seeks to understand the complex relationship among tumor cells, immune cells, fibroblasts, and endothelial cells, which together form the tumor mass (Hanahan and Coussens, 2012). In this section, we review the role of T and B lymphocytes in the TME and advocate for the utility of the zebrafish due to its high conservation in immune and oncogenic signaling cascades.

### The Innate Immune System as a Helper to Adaptive Immune Responses

Dendritic cells (DCs), macrophages, and neutrophils are the three major players in human innate immunity, all capable of infiltrating into the TME. DCs play a key role in presenting exogenous tumor antigens to activate cytotoxic CD8 + T cells for anti-tumor responses, with recent evidence indicating that the suppressive TME can dampen the anti-tumor response of DCs (Fu and Jiang, 2018). Macrophages and neutrophils have been categorized into two broadly defined groups: M1/M2 and N1/N2 (Fridlender et al., 2009). In general, M1 macrophages and N1 neutrophils are associated with TLR-mediated responses/interferon signaling and exert strong pro-inflammatory response against pathogens (Italiani and Boraschi, 2014; Murray, 2017). On the other hand, M2 macrophages and N2 neutrophils are typically linked with T regulatory cell (Tregs) responses and thus participate in cell proliferation and tissue repair (Italiani and Boraschi, 2014; Murray, 2017; Orecchioni et al., 2019; Wu et al., 2019). M1 macrophages and N1 neutrophils secrete inflammatory cytokines that can activate the adaptive immune system for anti-tumor responses (Selders et al., 2017). In contrast, M2 macrophages and N2 neutrophils secrete immunosuppressive cytokines (e.g., IL-10 and TGF-β) and further suppress the immune system by producing CCL22 to recruit Tregs (Fridlender et al., 2009; Gajewski et al., 2013). Macrophages and neutrophils can shift between the subtypes and thus impact the TME in vastly different manners. In the initial stages of tumor development, macrophages are believed to have a prominent M1 profile, characterized by NF-κB expression and capable of attacking the malignant cells (Mantovani and Sica, 2010). However, in clinically detectable tumors, the presence of tumor-associated macrophages (TAMs) predict worse patient outcomes for several types of cancer including those with breast and pancreatic origin (Bingle et al., 2002; Campbell et al., 2011; Zhang et al., 2012; Di Caro et al., 2016). Similarly, the presence of tumor-associated neutrophils (TANs) has been linked to poor prognosis across a wide range of cancers (Gentles et al., 2015). TAMs and TANs promote both tumor initiation and progression by enhancing angiogenesis, suppressing anti-tumor immunity, and facilitating the migration and invasion of tumor cells (Qian and Pollard, 2010; Argyle and Kitamura, 2018; Wu et al., 2019). TAMs are generally believed to possess a M2 profile due to their low expression of MHC class II (MHC-II) which reduces the adaptive immune response and increases production of angiogenesis-promoting elements such as vascular endothelial growth factors (Mantovani et al., 2006; Mantovani and Sica, 2010).

Dendritic cells, macrophages, and neutrophils have all been identified and characterized in zebrafish. Histochemical and ultrastructural analyses confirmed that zebrafish DCs possess the same morphological features and key canonical activities such as antigen-presentation to T cells as its mammalian counterpart (Lugo-Villarino et al., 2010). Because the expression of macrophage expressed gene 1 (*mpeg1*) is tightly restricted to macrophages in humans, transgenic lines, which express the fluorescent reporter genes under the promoter of *mpeg1*, have been developed to study macrophage-like cells in zebrafish (Ellett et al., 2011). For instance, the *Tg(mpeg1:mCherry*) line was crossed to the transgenic line driving *eGFP* expression under the tumor necrosis factor-alpha promoter to identify and visually track M1 (mCherry+; eGFP+) and M2 (mCherry+; eGFP-) macrophages in zebrafish (Nguyen-Chi et al., 2015). Moreover, upon induced inflammation through fin-wounding, tumor transplantation, or *Escherichia coli* inoculation, zebrafish M1 and M2 macrophages recapitulate the activation and gene expression patterns as established in higher vertebrates (Nguyen-Chi et al., 2015; Sanderson et al., 2015; Hasegawa et al., 2017; Nguyen-Chi et al., 2017; Tsarouchas et al., 2018). Similar to what is observed in humans, zebrafish neutrophils possess polymorphic nuclei, granules, and myeloid-specific peroxidase coupled with NADPH oxidase (Lieschke et al., 2001; Henry et al., 2013). Taken together, the presence of DCs, macrophages, and neutrophils in zebrafish and their similarities to humans make the zebrafish suitable to study innate immunity and their interactions with adaptive immune cells in the TME.

### T Lymphocytes

T lymphocytes are the major players in tumor immunity and include different subtypes characterized by their respective functions. Cytotoxic CD8 + T cells have become one of the most studied subtypes due to the recent success in checkpoint blockade therapies targeting CTLA-4 and PD-L1 signaling pathways (Gong et al., 2018). The anti-tumor response of CD8+T cells is canonically supported by CD4+Th1 helper cells (Ostroumov et al., 2018). Additionally, the TME also harbors other subtypes of CD4 + T lymphocytes, including Th2, Th17, and CD4 + /Foxp3 + Tregs that aid in immune evasion in most tumors (Speiser et al., 2016). T lymphocyte profiles within the TME of solid tumors vary greatly among patients. The ratio of one subtype versus another can predict treatment outcome and rates of disease relapse (Fridman et al., 2012). With similar subtypes and functions of T lymphocytes (Zhang and Wiest, 2016; Bajoghli et al., 2019), the zebrafish represents a useful model to facilitate understanding of the interplay among T lymphocyte subtypes within the TME. Translating this knowledge to the bedside can help improve immunotherapy and patient prognosis.

#### Cytotoxic CD8 + T Cells

Cytotoxic CD8 + T cells are derived from the αβ lineage of T-cell receptors (TCR) and recognize antigens presented by MHC-I molecules, serving as a major immune surveillance guard against tumors. Elevated numbers of activated CD8 + T cells within the TME are associated with positive outcomes among patients with breast cancer, colorectal cancer, renal cancer, and melanoma (Clemente et al., 1996; Tosolini et al., 2011; Gu-Trantien et al., 2013; Bohner et al., 2019; Ye et al., 2019). Cytotoxic CD8 + T cells recognize tumor antigens and physically engage tumor cells through spatial proximity to eliminate them (Pittet, 2009). Two main pathways are involved in this process: (1) granule exocytosis through the family of serine proteases: the perforin forms a pore on the membrane, allowing granule-associated enzymes (GZM) to access the target cytosol; or (2) apoptosis through cytotoxic effector ligands (e.g., TNFα or Fas) (Martínez-Lostao et al., 2015). However, as the TME becomes increasingly hostile over time, cytotoxic CD8 + T cells lose their ability to suppress tumor growth (Joyce and Fearon, 2015). For instance, lack of nutrients in the TME represents one disruptive factor, leading to exhaustion of these T cells (Chang and Pearce, 2016). Genetic alterations that deregulate oncogenic pathways, such as KRAS^*G*12*D*^ gain-of-function or *TP53* loss-of-function mutations, can result in the recruitment of large numbers of suppressive myeloid cells into the TME to inhibit cytotoxic T cells (Anderson et al., 2017). Thus, more research efforts are needed to understand the TME beyond what activates and mobilizes CD8 + cytotoxic T cells.

Cytotoxic CD8 + T cells have been characterized in teleosts and demonstrated similarities to their human counterparts. Using rainbow trout, analysis of CD8α + and CD8α− cells outside the thymus indicate the existence of CD4-/CD8 + and CD4 + /CD8− lymphocyte populations, which correspond to CD8 + cytotoxic cells and CD4 + Th or Tregs (Takizawa et al., 2011a). In addition, these CD8α + cells also expressed high levels of perforin and granulysin, indicating their effective cytotoxic function. Research conducted in Ginbuna carp again indicates the presence of CD8α + cells with perforin-mediated cytotoxic activity (Toda et al., 2009; Toda et al., 2011). Upon allogeneic insult, a novel granzyme was found upregulated on CD8 + cells in Ginbuna carp (Matsuura et al., 2014). Moreover, the cell-extrinsic apoptosis pathway through the Fas ligand has been detected in the zebrafish, but whether it is expressed in T cells is yet to be determined (Eimon et al., 2006). In Japanese flounder, the Fas ligand has been identified in T-like lymphocytes (Yamaguchi et al., 2019). Overall, cell-cell contact is a key characteristic of cytotoxic CD8 + T cells in the TME and this feature is observed in teleosts (Toda et al., 2011). CD8α + cells have been observed infiltrating the TME of salmon with intestinal tumors, which were induced from chronic gut inflammation (Bjørgen et al., 2019).

#### CD4 + Lymphocytes

The functions of CD4 + T lymphocytes in the TME are diverse and have been characterized to varying degrees. This can be attributed to the fact that most non-hematological tumors lack the expression of MHC-II molecules, which CD4 + T cells utilize for antigen presentation. Moreover, CD4 + T cells include various subtypes: Th1, Th2, Th17, and Tregs, which exert different and even opposite roles. In teleosts, there are two *cd4*-like paralogs, *cd4-1* and *cd4-2*. Their encoded proteins, which are widely coexpressed in zebrafish and rainbow trout (Yoon et al., 2015; Takizawa et al., 2016), differ in Ig domain structure. Cd4-1 exhibits a four Ig domain structure similar to mammalian CD4 (Castro et al., 2011). Cd4-2 has fewer Ig domains and its functional significance is currently unknown (Takizawa et al., 2016). Recent studies show that Cd4-1 + T cells in zebrafish infected with pathogens express Th1-, Th2-, and Th17-associated transcription factors and cytokines, indicating that T cell functions are well conserved in bony fish (Yoon et al., 2015; Maisey et al., 2016; Takizawa et al., 2016).

##### CD4 + Th1 Cells

The Th1 subtype produces large amounts of IFNγ, a cytokine that suppresses tumor growth by promoting proliferation and differentiation of CD8 + cytotoxic T cells and enabling the priming of APCs against tumor antigens (Shankaran et al., 2001; Huang et al., 2007; Ostroumov et al., 2018). Differentiation of Th1 cells is triggered by their exposure to the cytokines such as IFNγ and IL-12 and is characterized by the expression of the master transcription factor T-bet (Kanhere et al., 2012). IFNγ secreted by Th1 subset of cells can recruit natural killer cells and trigger cytotoxicity of tumor-infiltrating macrophages thus preventing tumor progression and angiogenesis (Haabeth et al., 2011; Kim and Cantor, 2014). This Th1 subset also aids in the clearance of pre-malignant, senescent hepatocytes working in conjunction with myeloid cells to prevent the development of liver cancer (Kang et al., 2011).

Both Th1 and Th2 subtypes are present in teleosts, similar to those observed in humans. T-bet, the transcription factor expressed in the Th1 subtype, has been identified and characterized in zebrafish (Mitra et al., 2010). Along with this, IFNγ, the characteristic cytokine of CD4 + Th1 cells, was identified for the first time outside of mammals in zebrafish (Igawa et al., 2006). Two experiments have been conducted in teleosts showing a generalized Th1-type response to antigen challenge. The zebrafish experiment showed that Cd4-1 + T cells, when exposed to a human antigen, induced T-bet and had higher expression of IFNγ (Yoon et al., 2015). A similar experiment conducted in rainbow trout revealed that Cd4 + lymphocytes expressed higher levels of IFNγ, IL-2, and IL-22 after being challenged with a bacterial pathogen (Takizawa et al., 2016). These results indicate a meaningful Cd4 + Th1 response in teleosts. Further research in zebrafish should seek to better understand Th1 cells beyond IFNγ production.

##### CD4 + Th2 Cells

The Th2 subtype secretes the cytokines IL-4, IL-5, and IL-13 and is characterized by the expression of the transcription factor GATA3 (Kanhere et al., 2012). Differentiation from naïve CD4 + into the Th2 subtype is regulated by IL-4 (Zheng and Flavell, 1997). Through studies in an airway hypersensitivity model, the transmembrane protein T cell immunoglobulin and mucin domain 1 (TIM-1) was found to be critical for Th2-type immune responses (Curtiss et al., 2012). Treatment with the TIM-1 antibody in the murine airway model resulted in the proliferation of Th2 cells, while the antibody against TIM-4, the ligand for TIM-1, induced T-cell proliferation in general (Meyers et al., 2005). Th2 cells can exert anti-tumor effects by recruiting both B cells and eosinophils into the TME (Nishimura et al., 1999; Mattes et al., 2003).

Compared to the Th1 subtype, Th2 cells in teleosts have been characterized with greater details. Dee et al. (2016) generated a zebrafish transgenic line, *Tg(cd4-1:mCherry)*, to monitor Cd4-1 + cells in different organs. The authors identified Th2-like cells in the gills, which express *gata3* and *il-4/13b*, consistent with what was found in salmonids (Takizawa et al., 2011b). They also found that Th2-like cells infiltrated into the TME of zebrafish melanoma. However, unlike the Th2 cells observed in the gills, these cells did not express *il-4/13b*, indicating heterogeneity of zebrafish Th2 subtype, a characteristic also observed in mammals. As previously seen in humans, blockade or knockdown of TIM-1 and TIM-4 in zebrafish significantly decreased the activation of Cd4 + T cells together with increased proliferation of Th2 subtype and B cells, indicating a key role of these two proteins in regulating CD4 + Th2 subtype (Xu et al., 2016).

##### CD4 + Th17 Cells

Differentiation from naïve CD4 + T cells into the Th17 subtype is positively regulated by the cytokines TGF-β, IL-6, and IL-23 (Zhu et al., 2010). Th17 cells express the transcription factor retinoid-related orphan receptor RORγt and produce the characteristic cytokines IL-17A, IL-17F, and IL-22. Th17 cells have been shown to induce auto-immune injury in tissues, a function opposite from Tregs (Bettelli et al., 2006). Although chronic inflammation is often carcinogenic, Th17 cells can exert powerful anti-tumor effects. A study using the murine model shows that Th17 polarized cells were highly effective in destroying tumors (Muranski et al., 2008). Additionally, adoptive T cell therapy utilizing Th17 polarized cells can induce cytotoxic CD8 + T cells (Martin-Orozco et al., 2009). In both studies, the anti-tumor effects of Th17 cells are even stronger than the control Th1 cells. Coupled with their long-lived and stem cell-like nature, Th17 cells are critical in anti-tumor responses (Muranski et al., 2011).

Evidence supports the presence of Th17 cells in teleosts, particularly in zebrafish. Firstly, the ROR family is present in zebrafish, including the RORγt transcription factor critical for Th17 cell differentiation (Flores et al., 2007; Monte et al., 2012; Zhang et al., 2013). Five different forms of IL-17 are also found in zebrafish, and they are upregulated in organs of the adaptive immune system such as the spleen and kidney marrow upon lipopolysaccharide (LPS) stimulation (Gunimaladevi et al., 2006). Transcriptome profiling analysis revealed that zebrafish vaccinated with an attenuated bacterial pathogen upregulated key Th17 cytokines, such as IL-17A and IL-22 together with RORγt (Zhang et al., 2013). This demonstrates a clear causal relationship between antigen challenges and the expression of these characteristic Th17 markers. Interestingly, large numbers of Th17-like cells expressing *il17a/f1*, *il17a/f3*, *il22*, and *rorca* are found recruited to the gut of a zebrafish model of autoimmune and inflammatory diseases, confirming their recognition of self-antigens (Coronado et al., 2019).

##### CD4 + Tregs

Tregs, another subtype of CD4 + T cells, produce immunosuppressive cytokines such as TGFβ and IL-10, and is characterized by the expression of the transcription factor Foxp3a (Kim and Cantor, 2014; Speiser et al., 2016). Tregs normally prevent excessive autoimmune responses and promote wound healing. In the TME, they suppress CD8 + cytotoxic T cells and support angiogenesis and metastasis of tumors (Zou, 2006). In fact, a significant proportion of tumor-infiltrating CD4 + cells are Tregs (Kim and Cantor, 2014). A high ratio of Tregs to cytotoxic CD8 + T cells is correlated with poor prognosis of patients with multiple cancer types, including pancreatic cancer, ovarian cancer, and colorectal cancer (Preston et al., 2013; Tang et al., 2014; Bencsikova et al., 2019). Hence, the key to the treatment of solid tumors is to suppress the recruitment of Tregs to the TME or to inhibit their immunosuppressive functions.

It has been demonstrated that Foxp3a*-*expressing Treg-like cells exist in zebrafish (Quintana et al., 2010; Kasheta et al., 2017). Along with a transgenic line that marks out these Treg-like cells by expressing eGFP-fluorescent reporter gene under the zebrafish *foxp3a* promoter, a mutant zebrafish line with loss-of-function of *foxp3a* has also been generated (Kasheta et al., 2017). Using these tools, Kasheta et al. (2017) found that *foxp3a-/*- mutant zebrafish exhibit overall inflammation in tissues, resembling severe human autoimmune disorders. Using the *Tg(cd4-1:mCherry)* transgenic zebrafish, Dee et al. (2016) demonstrated the existence of Treg-like cells in the gut mucosa. Research studying Tregs’ function in organ regeneration has revealed many potential roles of Tregs in tumorigenesis. When zebrafish were used to model spinal cord, heart, and retina regeneration, CD4 + Treg -like cells were found to quickly migrate to the damaged areas to aid in tissue regeneration (Hui et al., 2017). The ablation of these cells significantly impaired the regenerative capabilities of the tissue (Hui et al., 2017).

#### TCRγδ T Cells

The decision between the αβ and γδ fates is one of the first made by T cell progenitors in the thymus. The two groups diverge from the same progenitors based on the strength of TCR signaling: with the strong one generating γδ cells and the weak one generating αβ cells (Ciofani and Zuniga-Pflucker, 2010; Wong and Zuniga-Pflucker, 2010; Zarin et al., 2015). αβ cells are defined by successful rearrangement of the TCRβ loci and their progression through a CD4/CD8 double-positive stage, while the γδ subtype rearranges the TCRγ and TCRδ loci and avoids this double-positive stage (Kreslavsky et al., 2010; Zarin et al., 2015). The TCRαβ lineage comprises the vast majority of T cells in the body, while only 2–10% of the total T lymphocytes exhibit γδ characteristics (Sheridan et al., 2013). Unlike CD4+ or cytotoxic CD8 + T lymphocytes, these rare γδ T cells are not restricted to antigens presented by a particular class of MHC molecules and possess both cytotoxic and immune-stimulating properties. For instance, they express cytotoxic ligands (e.g., FasL) and have capacities of both phagocytosis and antigen presentation (Lawand et al., 2017; Zhao et al., 2018). Indeed, the high frequency of γδ cells is associated with positive prognostic outcomes across 25 different types of cancer, especially among solid tumors (Gentles et al., 2015). This association was even stronger than that observed for cytotoxic CD8 + T cells, highlighting the anti-tumor effect of γδ cells in the TME (Gentles et al., 2015).

Distinctive γδ T cells have been observed in teleosts and characterized in zebrafish. The TCRγ locus has been identified in the zebrafish genome assembly using conserved elements among species (Seelye et al., 2016). Wan et al. (2017) found that zebrafish γδ T cells exhibit the characteristic CD4-; CD8 + surface markers with similar flow cytometry scatter patterns/morphology as seen in human γδ cells. Moreover, both the non-specific phagocytic and antigen-presenting characteristics are also preserved in the zebrafish (Wan et al., 2017). The presence of TCRγ was also found in the gut mucosa of other teleosts, such as the sea bass (Picchietti et al., 2011). The above evidence indicates that the zebrafish is suitable for in-depth studies of γδ T cells in the TME.

### B Lymphocytes

The role of B cells in the TME is less understood compared to T cells. Across all subtypes of breast cancer, nearly 60% of tumor-infiltrating lymphocytes within the TME were found to be B cells (Coronella-Wood and Hersh, 2003). A recent study, which examined the prognostic significance of tumor-infiltrating B and plasma cells, showed that both types were associated with either positive or neutral outcomes across a wide array of solid-tumor cancer types, including lung, colorectal, gastric, ovarian, and hepatocellular cancers (Wouters and Nelson, 2018). However, when the disease stage was taken into consideration for oro- and hypopharyngeal cancer, B cells were associated with a positive prognosis in patients with early disease but a negative prognosis for those with advanced disease (Wouters and Nelson, 2018).

Different subtypes of B cells can have opposite roles in the TME, either tumor-promoting or suppressing (Shen et al., 2016; Yuen et al., 2016; Largeot et al., 2019). Human mature B cells in the periphery include two main groups: follicular and marginal zone B cells. Follicular B cells can differentiate into IgG, IgE, and IgA antibody-secreting cells (ASCs) with the help of T cells, and can also form IgM ASCs in a T-cell independent manner (Allman and Pillai, 2008). Marginal zone B cells generally serve in an innate-like capacity, while pathogenic triggers such as LPS can induce them to become short-lived plasma cells (Allman and Pillai, 2008). In addition, specific subtypes of B cells expressing CD19, CD20, CD11c, and B220 can also function as APCs (Largeot et al., 2019). Beyond antigen presentation, CD19 + B cells can also express the death ligand FasL to directly induce cytotoxicity when stimulated by IL-17A (Lu et al., 2016). These same cells are negatively regulated by IL-10 (Tao et al., 2017). IL-21 can also stimulate B cells within the TME to produce granzyme B (Jahrsdörfer et al., 2006). Finally, B regulatory cells (Bregs) are a small population of B cells participating in immunomodulation. They exert immunosuppression by enhancing the activity of Tregs, secreting immunosuppressive cytokines such as IL-10, and suppressing the effector CD4+ and CD8 + T cells via the production of TNFα (Olkhanud et al., 2011; Sarvaria et al., 2017). Evidence from multiple studies indicates that Bregs are capable of shielding cancers from the immune system (Schioppa et al., 2011; Murakami et al., 2019). Bregs have also been shown to induce tissue heterogeneity in melanoma through crosstalk between tumor-produced fibroblast growth factor 2 and B-cell origin insulin growth factor 1 to reduce the effectiveness of kinase-inhibitor therapies (Somasundaram et al., 2017).

B cells have been characterized in zebrafish. While IgM and IgD are traditionally considered as the main surface markers for B cells in teleosts, another B cell marker, IgZ, was recently identified in zebrafish immune tissues such as the kidney, spleen, and gills after the LPS challenge (Hu et al., 2010). Page et al. (2013) characterized the development and behavior of IgM + B cells in zebrafish. Utilizing three fluorescent transgenic lines, they defined the existence of pro-B (Pax5^+^Rag2^+^IgM^–^) and immature/mature (Pax5^+^Rag2^–/lo^IgM^+^) B cells in the kidney marrow of adult zebrafish (Page et al., 2013). Furthermore, they characterized plasma B cells and discovered a population of CD45-Blimp1 + cells that express plasma-based characteristic markers, such as *xbp1*, *cd40*, and *irf4* (Page et al., 2013). Liu et al. applied a fluorescent *cd79/cd79a* transgenic reporter line to show that the pre-B cell stage does not exist in zebrafish, a key difference in B cell development between zebrafish and humans (Liu et al., 2017). Further work indicates that CD79a and CD22 can serve as meaningful markers to distinguish multiple stages of B cell development in teleosts (Liu et al., 2017; Peñaranda et al., 2019). Research examining gastrointestinal tumors in salmonids detected infiltration of IgM + B cells in the tumor stroma and also in metastatic outgrowths in the liver, mirroring what was observed in certain human cancers (Bjørgen et al., 2019). The above evidence supports the suitability of the zebrafish for studying B cell immunity in the TME.

## Technological Advances for the Zebrafish

Among vertebrate models, the zebrafish possesses unique advantages for tumor immunity research. The high fecundity of female zebrafish provides hundreds of progeny ideal for statistical analysis, while their external reproduction provides unprecedented access to study the early development of the immune system and cancer (Zon and Peterson, 2005). The optical clarity of embryos and juvenile fish together with the development of highly transparent *Casper* adult fish enables live imaging studies of tumor-immune cell interactions. Moreover, significant advances have been made over the years to develop the technology needed for zebrafish research, including an assortment of transgenesis and mutagenesis techniques for gene modulations, as well as the ability to conduct large-scale genetic and chemical screens using zebrafish (Suster et al., 2009).

### Transgenesis

Early transgenic zebrafish were created by microinjecting linearized plasmid DNA into one-cell-stage embryos (Stuart et al., 1988; Amsterdam et al., 1995). However, this technique was limited by low rates of transmission of the transgene to the progeny (Suster et al., 2009). In recent years, the development of meganuclease or transposon-mediated transgenesis has led to the generation of stable transgenic zebrafish (Michailidou et al., 2009; Chen et al., 2014). Furthermore, the application of conditional transgenesis allows for the inducible expression of target genes, such as oncogenes like *xmrk* and *kras^*G*12*D*^* (Li et al., 2012; Enya et al., 2018). One example is the Cre/lox system, which can be combined with the GAL4/Upstream activating system (GAL4/UAS) to regulate oncogene expression under tissue-specific promoters (Santoriello et al., 2010; Enya et al., 2018; Park and Leach, 2018). Another example is the LexPR system, which initiates the transcription of a specific reporter gene through binding to an operon (Kenyon et al., 2018). Finally, the Tet-On system allows the spatial induction of an oncogene under a tissue-specific promoter (Li et al., 2012). The combination of zebrafish transgenesis and its prolificacy has led to the generation of numerous transgenic lines rapidly in a cost-effective manner.

### Targeted Gene Inactivation and Genome Editing

Gene inactivation and editing provide another means to modify gene expression in zebrafish. Endonucleases such as transcription activator-like effector nucleases have been utilized to induce targeted double-stranded DNA breaks in tumor suppressor genes to trigger tumorigenesis (Sander et al., 2011; Dahlem et al., 2012; Ignatius et al., 2018). Another approach to inactivate genes in zebrafish is the application of zinc-finger proteases to induce double-stranded breaks followed by non-homologous base-pairing (Payne and Look, 2009). Recently, the cluster regulatory interspaced short palindrome repeats CRISPR/Cas9 system has become the major method for zebrafish gene editing. This advanced technique can induce double-stranded DNA cleavages to delete DNA sequences or introduce genetic modifications in highly targeted locations within the genome. The high efficacy of the CRISPR/Cas9 system, combined with the ease of microinjection of the one-cell-stage embryos, allows for simultaneous targeting of multiple genes in zebrafish to provide insights into how each lineage develops. Furthermore, CRISPR/Cas9 manipulates the genome in a highly controlled manner with up to 50% accuracy (Liu et al., 2019). Collectively, these techniques have enabled the efficient procurement of genetic editing in zebrafish.

### Xenografts

Xenografts are animals injected with human cells. While the murine xenografts take weeks or even months to establish, it only takes days to weeks to establish zebrafish xenograft (Xiao et al., 2020). Zebrafish are often transplanted as embryos with fluorescently labeled tumor cells, including patient-derived cancer cells (Au-Kemp et al., 2009; Veinotte et al., 2014; Hason and Bartůněk, 2019; Vargas-Patron et al., 2019). Due to the absence of the adaptive immune system at the early embryonic stage, tumor cell engraftment can occur in zebrafish easily, generating multiple models to study metastasis of human tumor cells including those from patients (Marques et al., 2009; Cabezas-Sainz et al., 2018; Xu et al., 2018). A recent example shows that zebrafish xenografts can serve as a fast, cost-effective preclinical tool with live imaging capabilities to investigate CAR T cell-mediated killing of human cancer cells in B cell malignancies (Pascoal et al., 2020). Although zebrafish are normally reared at an optimal temperature of 26–28°C, xenografted embryos can tolerate an upper limit of 37°C to simulate conditions within the human body (Cabezas-Sainz et al., 2018; Morgan et al., 2019; Yan et al., 2019). Despite the minimal effect on the proliferation of xenografted cells (Cabezas-Sainz et al., 2018; Xu et al., 2018), it is unknown whether these cells experience higher rates of mortality. A more detailed characterization is required to fully understand the properties of these transplanted tumor cells. Additionally, the field will benefit from fine-tuning husbandry techniques to improve zebrafish tolerance of higher temperatures.

For the adult zebrafish, human tumor cells can be introduced through intraperitoneal injection (Patton et al., 2005). However, successful transplantations can only occur in the immunocompromised zebrafish (Vargas-Patron et al., 2019). Similar to mammals, lymphocytes and APCs in adult zebrafish recognize the transplanted tumor as foreign cells and eliminate them (Langenau et al., 2004; Dee et al., 2016). APCs present tumor-antigens through MHC-II molecules to activate Cd4 + T cells and initiate anti-tumor immune responses (Wittamer et al., 2011; Dee et al., 2016). Hence, T cells must be ablated in recipient zebrafish through irradiation, chemical treatments, or mutagenesis to ensure engraftment (Langenau et al., 2004). To facilitate the use of zebrafish for cancer research, a panel of immunocompromised zebrafish mutants in the transparent *Casper* zebrafish background have been generated over the past few years (Table 3) (Moore et al., 2016; Tang et al., 2017). The zebrafish xenografts allow for therapeutic testing of compounds in a rapid and cost-effective manner. Moreover, coupled with the imaging technique, zebrafish xenografts represent valuable tools to study human tumor cell behavior, including proliferation, apoptosis, invasion, and interaction with host cells (Zhao et al., 2011; Jung et al., 2012; Drabsch et al., 2013; Pudelko et al., 2018; Vargas-Patron et al., 2019).

### Small Molecule Screens

Due to their high fecundity and small size, zebrafish are suitable for small molecule screens to identify anti-cancer compounds, and zebrafish xenografts can even be used to screen for personalized therapeutics (Rennekamp and Peterson, 2013; Veinotte et al., 2014). The rapid development of zebrafish allows for the simultaneous assessment of drug efficacy and toxicity in a high-throughput manner (Williams and Hong, 2016; Hason and Bartůněk, 2019; Yan et al., 2019). One successful example of using zebrafish xenografts for small molecule screening is the identification of the drug, clotrimazole, for the treatment of melanoma (Precazzini et al., 2020). This drug, when co-administered with specific inhibitors targeting oncogenes such as Ras expression in melanoma, blocked transformed malignant cells from proliferating (Precazzini et al., 2020). Other researchers have also utilized zebrafish for small molecule screens to identify compounds that disrupt specific biological pathways important to cancer initiation, progression, or maintenance (Gore et al., 2018; Letrado et al., 2018; Lam and Peterson, 2019). Along with the discovery of novel oncogenic pathways that these compounds target, zebrafish chemical genetics have led to an improved understanding of tumor progression and treatment resistance (Gallardo et al., 2015; Dang et al., 2016).

### Advanced Imaging Techniques

The optical clarity of the zebrafish during the embryonic to juvenile stage allows for high-resolution imaging. The generation of *Casper* fish, which lack pigmentation and have thin muscle tissues, extended this imaging capacity to adult zebrafish (White et al., 2008; Blackburn et al., 2011). A broad spectrum of fluorescent proteins can be exploited for differential labeling and visualization of stromal and tumor cells, including vasculatures and different types of immune cells (Oralova et al., 2019). Live imaging in zebrafish unfolds the process of neovascularization, morphology of tumor cells, and their movement, as well as the dynamic interactions with their surrounding tissues (Blackburn et al., 2011; Ignatius and Langenau, 2011). Low-resolution fluorescent macroscopes equipped with different LED lights and filters enable both still imaging and live-video recording of up to 30 zebrafish simultaneously (Blackburn et al., 2011). Laser confocal microscopy provides images with high resolution and contrast, but images are procured one pixel at a time, leading to photo-toxicity over time (Ignatius and Langenau, 2011). A spinning-disc confocal microscope that captures multiple pixels at once can ameliorate these drawbacks. Alternatively, the two-photon microscopy allows for time-lapse images of deep cell structures at high-resolution with long exposure times (Ignatius and Langenau, 2011).

### Genetic Screens

Over the past two decades, a large body of work on zebrafish has been focused on genetic screens, uncovering numerous genes critical for tumorigenesis. The genetic screens were either based on chemical or insertional mutagenesis. For the former, the chemical ENU was often used as the mutagen, leading to the discovery of many zebrafish mutant lines for cancer research (Driever et al., 1996; Haffter et al., 1996; Shive et al., 2010). On the other hand, insertional mutagenesis screens utilize exogenous DNA or retroviruses as the mutagens (Sivasubbu et al., 2007). An additional screening method exploited by zebrafish is target-induced local lesions in genomes, a reverse genetic strategy that can identify point mutations of the gene of interest (Da Costa et al., 2014). More recently, screens through transgenic overexpression of genes, such as those using the MiniCoopR vectors, have been conducted to identify genetic modifiers of a known tumor phenotype (Iyengar et al., 2012; Ablain et al., 2018). All of these screening methods have led to the generation of a panel of zebrafish mutants that develop tumors and the identification of novel tumor suppressor genes, oncogenes, and genes that modify the existing tumor phenotype.

## Available Resources Enabling Zebrafish for Tumor Immunity Research

Along with the technological advances, there are a number of resources suitable for tumor immunity research using the zebrafish. Among them, transgenic fluorescent reporter lines help label and track different types of immune cells, especially those participating in adaptive immunity (Table 1). In addition, researchers have also generated and validated a panel of antibodies recognizing zebrafish T and B cells as well as mutant lines in which these adaptive immune cells are depleted (Tables 2, 3). Finally, two dozen zebrafish models of cancer have already showcased the power of this organism in understanding disease etiologies (Table 4). Together, these valuable resources opened the door for researchers to probe specific interactions between immune and tumor cells in various types of cancers, and to expand our understanding of the TME and adaptive tumor immunity.

**TABLE 1:** Transgenic fluorescent reporter lines for tracking adaptive immune cells.

Promoter	Fluorophores	Possible cell type labeled	References
*lck*	*eGFP*; *dsRed*	Lymphoid progenitors (T/B cells, NK cells, dendritic cells)	Langenau et al., 2004; Lugo-Villarino et al., 2010; Borga et al., 2019; Carmona et al., 2017
*ikaros*	*eGFP*	Lymphoid progenitors	Bajoghli et al., 2009
*rag2*	*mCherry*	Lymphoid progenitors (T/B cells)	Langenau et al., 2003
*rag1*	*eGFP*	Lymphoid progenitors (T/B cells)	Jessen et al., 1999
*cd4*	*mCherry*	CD4 + T cells and macrophages	Dee et al., 2016
*foxp3a*	*eGFP*, *RFP*	T regulatory cells	Hui et al., 2017
*mhc2dab*	eGFP	B-cells/myeloid cells	Wittamer et al., 2011
*cd45*	*dsRed*	T-cells/myeloid cells	Wittamer et al., 2011
*IgM*	*eGFP*	Mature IgM + B cells/plasma cells	Page et al., 2013
*cd79a*	*eGFP*	Persistent B cell marker beginning at pro-B cells	Liu et al., 2017
*cd79b*	*eGFP*	Marker for B cells beginning at pro-B but less effective for mature B cells	Liu et al., 2017

**TABLE 2:** Antibodies recognizing markers of zebrafish immune cells.

Antigen	Type	Reactive species	Host species	Isotype	References
CD4-1	Monoclonal	Zebrafish, Gibuna crucian carp	Rat	IgG2a	Miyazawa et al., 2018
CD8α	Monoclonal	Zebrafish, Gibuna crucian carp	Rat	IgG2a	Miyazawa et al., 2018
CD4-1	Polyclonal	Zebrafish	Rabbit	IgG	Yoon et al., 2015
TCR-α	Polyclonal	Zebrafish	Rabbit/mouse	IgG	Wan et al., 2017
TCR-β	Polyclonal	Zebrafish	Rabbit/mouse	IgG	Wan et al., 2017
TCR-γ	Polyclonal	Zebrafish	Rabbit/mouse	IgG	Wan et al., 2017
TCR-δ	Polyclonal	Zebrafish	Rabbit/mouse	IgG	Wan et al., 2017
CD154	Polyclonal	Zebrafish	Rabbit/mouse	IgG	Gong et al., 2009
CD40	Polyclonal	Zebrafish	Rabbit/mouse	IgG	Gong et al., 2009
IgM	Polyclonal	Zebrafish	Rabbit/mouse	IgG	Gong et al., 2009
CD80/86	Polyclonal	Zebrafish	Rabbit/mouse	IgG	Zhu et al., 2014
CD83	Polyclonal	Zebrafish	Rabbit/mouse	IgG	Zhu et al., 2014
CD4	Polyclonal	Zebrafish	Rabbit	IgG	Zhu et al., 2014
Lcp1	Polyclonal	Zebrafish	Rabbit	IgG	Redd et al., 2006

**TABLE 3:** Immunodeficient zebrafish mutants.

Genotype	Description	Background of fish	ZFIN ID	References	Access
*rag1^*t*26683^*	No T cells, no B cells	AB	ZDB-FISH-150901-17632	Wienholds et al., 2002; Tokunaga et al., 2017	Nüsslein-Volhard Lab
*rag2^*E*450*fs*^*	No T cells, low B cells	AB	fb101	Tang et al., 2014	Langenau lab
*rag2^*E*450*fs*^*	No T cells, low B cells	Casper	fb101	Tang et al., 2016	Langenau lab
*jak3 ^*P*369*fs*^*	No T cells, no NK cells	Casper	fb102	Moore et al., 2016	Langenau lab
*prkdc ^*D*3612*fs*^*	No T or B cells	Casper	fb103	Moore et al., 2016	Langenau lab
*il2rga ^*Y*91*fs*^*	No T cells or NK cells	Casper	fb104	Tang et al., 2017	Langenau lab
*il2grb ^*I*30*fs*^*	Not characterized	Casper	Pending	Pending	Langenau lab
*zap70 ^*Y*442*fs*^*	No T cells	Casper	y442	Moore et al., 2016	Langenau lab
*prkdc*^*fb103/fb103*^	No T, B, or NK cells	*Casper*	Pending	Yan et al., 2019	Langenau lab
*il2rga*^*fb104/fb104*^					
*c-myb* (t25127)	No myeloid, erythroid, or lymphoid cells			Soza-Ried et al., 2010	Boehm lab

**TABLE 4:** Zebrafish cancer models.

Cancer type	Technology used to generate tumor phenotype	Genotype	Fish background	Cell type	References
Peripheral nerve-sheath tumor (PNST), angiosarcoma, germ cell tumors, leukemia	TALENs	*tp53*^*del/del*^	*CG1 syngeneic*	Multiple	Ignatius et al., 2018
PNST, rhabdomyosarcoma, myeloproliferative disorder, intestinal hyperplasia	Heat-shock induced Cre-Lox	β-*actin:LoxP-EGFP-LoxP-kRASG12D*; *hsp70:Cre*	WT		Le et al., 2007
Epithelioid sarcoma, angiosarcoma, undifferentiated pleomorphic sarcoma	CRISPR-CAS9	*atrx ^+/del^*	*tp53*^*del/del*^,*nf1^*del/del*^*	Multiple	Oppel et al., 2019
Melanoma	Tol2 transgenesis	*mitfa:BRAF^*V*600*E*^*	*tp53^*M*214*K*^*	Melanocytes	Patton et al., 2005
	Tol2 transgenesis	*mitfa:EGFP-NRAS^*Q*61*K*^*	*tp53^*M*214*K*^*		Dovey et al., 2009
	I-SceI meganuclease	*mitfa:HRAS^*G*12*V*^*; *mitfa:eGFP*	WT		Michailidou et al., 2009
	I-SceI meganuclease	*mitfa:HRAS^*G*12*V*^*; *mitfa:mCherry*	WT		Michailidou et al., 2009
	Inducible LexPR	*mitfa:LexPR-Cerulean x Crys*β*:eCFP-LexOP:mCherry-NRas^*Q*61*K*^*	WT		Kenyon et al., 2018
	Gal4-UAS	*kita:GalTA4*; *UAS:mCherry*; *UAS:eGFP-HRAS^*G*12*V*^*	WT		Santoriello et al., 2010
	Inducible LexPR	*kita:LexPR-Cerulean x Crys*β*:eCFP-LexOP:mCherry-NRas^*Q*61*K*^*	WT		Kenyon et al., 2018
Non-melanoma skin cancer	Tol2 transgenesis	*krt4:c-myc^*T*58*A*^*; *cdc6:mCherry*	WT	Epidermal cells	Chen et al., 2014
Hepatocellular carcinoma	Tol2 transgenesis	*fabp10a:RPIA*; *myl7:GFP*		Hepatocytes	Chou et al., 2019
	Tol2 transgenesis	*fabp10:rtTA2sM2*; *TRE2:eGFP-krasG12V*			Chew et al., 2014
	Tol2 transgenesis	*fabp10:eGFP-kras^*V*12^*			Nguyen et al., 2011
	Inducible Tet-On	*fabp10:TA*; *TRE:Myc; krt4:GFP*			Li et al., 2012
	Inducible Tet-On	*fabp10:TA*; *TRE:xmrk*; *krt4:GFP*			Li et al., 2012
	Inducible LexPR	*fabp10:LexPR*; *LexA:eGFP x*; *cryB:mCherry*; *LexA:eGFP-kras^*V*12^*			Nguyen et al., 2012
Familial adenomatous polyposis/hereditary non-polyposis colon cancer	Gal4/UAS	*pInt-Gal4*;*5x UAS:eGFP-P2A-kras^*G*12*D*^*	WT	Intestinal epithelial cells	Enya et al., 2018
	Inducible LexPR	*pDs-ifabp:LexPR-Lexop:eGFP-kras^*V*12^*	WT		Lu et al., 2018
Glioblastoma	Inducible Tet-On	*gfap:rtTA*; *TRE:mCherry-KRAS^*G*12*V*^*	WT	Glial cells	Ju et al., 2015
	Inducible Tet-On	*krt5:rtTA*; *TRE:mCherry-KRAS^*G*12*V*^*	WT	Skin epithelial cells/Glial cells	Ju et al., 2015
Peripheral nerve-sheath tumor (PNST)	Tol2 transgenesis	*Tg(-8.5nkx2.2a:GFP)^*ia*2^*	WT	Peripheral nerves	Astone et al., 2015
Rhabdomyosarcoma	Tol2 transgenesis	*rag2:KRAS^*G*12*D*^*; *rag2:dsRed2*	WT	Myoblasts	Langenau et al., 2007
Costello syndrome	Inducible heat-shock	*hsp70:GFP-HRAS^*V*12^*	WT	Constitutive	Santoriello et al., 2009
Thyroid cancer	Tol2 transgenesis	*tg:BRAF^*V*600*E*^-pA*; *tg:TdTomato-pA*	WT	Thyrocytes	Anelli et al., 2017
Neuroblastoma	Tol2 transgenesis	*dβh:eGFP-MYCN*	WT	Neuroblasts	Zhu et al., 2012
		*dβh:eGFP*; *dβh:MYCN*	WT		Tao et al., 2017
		*d*β*h:c-MYC*; *d*β*h:mCherry*	WT		Zimmerman et al., 2018
Pancreatic ductal adenocarcinoma	BAC recombineering	*ptf1a:eGFP-KRAS^*G*12*V*^*	WT	Pancreatic progenitor cells	Park et al., 2008
	Gal4/UAS	*ptf1a:Gal4*; *UAS:eGFP-KRAS^*G*12*D*^*	WT		Schiavone et al., 2014
	Gal4/UAS and Cre/Lox	*ptf1a: CRE^*ERT*2^*; *LSL-GAL4*; *UAS-KRAS^*G*12*V*^*	WT		Park and Leach, 2018
Pituitary adenomas	Tol2 transgenesis	*Pomc:Pttg*; *POMC:eGFP*	WT	Corticotrophs	Liu et al., 2011
Testicular neoplasias	ENU forward screen	*brca2^*Q*658*X*^*	N/A	Spermatogonia	Shive et al., 2010

### Transgenic Lines for Tracking Adaptive Immune Cells

Transgenic fluorescent reporter lines represent powerful tools to track immune cells and their behaviors in the TME (Table 1). As the conserved genes governing the development of the adaptive immune system are identified, multiple fluorescent reporter lines have been rapidly generated to mark different immune components in zebrafish using their respective promoters. Similar to the reporter lines for the innate immune system, such as *Tg(mpeg1:eGFP)* for macrophage and *Tg(mpx:GFP)* for neutrophils (Ellett et al., 2011; Wang et al., 2014), reporter lines for the adaptive immune system have been utilized to understand the development of lymphocytes in vertebrates (Table 1) (Willett et al., 1999; Langenau et al., 2004). Since these reporter lines can label both lymphoid progenitors and mature lymphocytes, they represent valuable tools to investigate the role of T and B cells in the context of solid tumors and their differentiation in the TME. Interestingly, recent work has found that eGFP fluorescence levels in the *Tg(lck:eGFP)* line can distinguish B or T cells, indicating the usefulness of this transgenic line in simultaneously studying the behaviors of T and B cells (Borga et al., 2019). The *Tg(cd4-1:mCherr*y) line can label both Cd4 + lymphocytes and macrophages, and has been used to document the conservation of zebrafish lymphocyte development and oncoimmunophenotypes (Dee et al., 2016).

### Antibodies for the Study of the Adaptive Immune System in Zebrafish

The development of zebrafish antibodies is an ongoing process (Table 2). Prominent work from the Shao lab at the Zhejiang University has generated a wide variety of zebrafish antibodies against a number of key components in the adaptive immune system, such as IgM, CD40, TCRα/β/γ/δ, CD80/86, and CD83 (Gong et al., 2009; Zhu et al., 2014; Wan et al., 2017). In addition, cross-reactive antibodies developed for other species have helped expand this toolbox. For instance, CD4-1 and CD8α antibodies developed for Ginbuna carp have been validated for successful use in zebrafish (Miyazawa et al., 2018).

### Immunocompromised Zebrafish Mutants

Immunocompromised zebrafish serve as the ideal host for tumor cell transplantation and are useful tools that help dissect the contribution of specific immune cells to tumorigenesis (Table 3). This line of research began with the development of the *rag1-/-* mutant (Wienholds et al., 2002), followed by the identification of *c-myb*(t25127) mutant fish (Soza-Ried et al., 2010). The Langenau laboratory later developed additional mutant fish with excellent survivability, leading to the establishment of multiple human cancer xenografts in which patterns of angiogenesis and metastasis are studied (Tang et al., 2014; Moore et al., 2016; Tang et al., 2016; Tang et al., 2017; Yan et al., 2019). Combined with their optical clarity, these immunocompromised zebrafish allows for the visualization of single-cell engraftments and the tracking of cancer cell proliferation and migration (Yan et al., 2019). In addition, when transplanted with human cancer cells at the adult stage, these mutant zebrafish are instrumental in evaluating and screening for compounds for personalized application (Hason and Bartůněk, 2019; Yan et al., 2019).

### Zebrafish Models of Human Cancers

Beyond xenograft models mentioned above, zebrafish genetic models for human cancers represent one area with the most growth over the past 20 years. Pairing appropriate promoters with cancer-prone genetic alterations have led to the development of over two dozens of zebrafish cancer models (Table 4). Most of these models were generated through oncogene overexpression. Among them, the solid tumor models, such as melanoma and neuroblastoma, are well suited for investigating tumor-immune cell interactions *in vivo*. Examples of this type of research include studying the role of myeloid cells in early melanoma development utilizing the BRAF^*V*600*E*^ transgenic fish as well as the LexPR-regulated conditional *NRAS^*Q*61*K*^* zebrafish (Patton et al., 2005; Kenyon et al., 2018). The zebrafish expressing either N-MYC or C-MYC under the adrenal promoter *dβh* to model neuroblastoma recapitulates human phenotypes (Zhu et al., 2012; Tao et al., 2017; Zimmerman et al., 2018). C-MYC was also paired with a different promoter to generate a zebrafish model of hepatocellular carcinoma (Li et al., 2012).

Zebrafish models of human cancers have also been generated through inactivating tumor suppressors. An example of this is the zebrafish with *tp53* mutations that primarily develop malignant peripheral nerve sheath tumors (MPNSTs) (Berghmans et al., 2005). Interestingly, a recent development of the zebrafish with deletion of *tp53* revealed tumor development ranging from MPNSTs, angiosarcomas, and germ cell tumors to an aggressive natural killer cell-like leukemia (Ignatius et al., 2018). Since this model was generated in the CG1 syngeneic zebrafish strain, researchers can easily transplant tumors into this zebrafish to visualize metastatic and angiogenic capacities of tumor cells (Ignatius et al., 2018). CRISPR/Cas9 technology has also been used to delete tumor suppressor genes such as ATRX (Oppel et al., 2019). When ATRX was knocked out in the *tp53* mutant fish background, the fish developed epithelioid sarcoma, angiosarcoma, and undifferentiated pleomorphic sarcoma (Oppel et al., 2019). ENU screens generated additional cancer models, such as the *brca2^*Q*658*X*^* mutant fish that develop testicular neoplasias (Shive et al., 2010). The availability and conservation of these zebrafish cancer models further expand the toolbox of the zebrafish for tumor immunity research.

## Proof of Principle: A Small Fish DIP

Both genetic and xenograft cancer models of zebrafish are suitable for tumor immunity studies. The use of genetic models of cancer provides insights into how the immune system responds to malignant transformation and progression during animal development. The fact that zebrafish share conserved telomere regulation pathways and also live in a non-sterile environment as humans provides unique advantages for its utility in studying tumor immunity in comparison to mice (Carneiro et al., 2016). Xenograft assays, ranging from the transplantation of cultured human cell lines to primary patient cells, can complement the genetic models to quickly validate immunotherapies and other selected agents that can kill cancer cells but not the developing fish. In addition, some descriptive studies of the TME in teleosts have been conducted when cancer development is triggered by diet-mediated inflammation, such as metastatic intestinal adenocarcinoma (Bjørgen et al., 2019).

### Zebrafish Genetic Models to Probe Tumor-Immune Interactions

Zebrafish genetic models of cancer are often paired with the fluorescent reporter lines to differentially label both the tumor and the immune system, especially the adaptive immune components (Tables 1, 4). Combined with the imaging capacities of the zebrafish, interactions between tumor and specific immune cells arising from the fish can be tracked and studied with high fidelity as seen in human cancers. Dee et al. studied the infiltration of melanoma by T cells and their findings recapitulate what is observed in mammalian systems (Dee et al., 2016). When melanoma develops from the radial growth phase into nodular tumors, T cell activities reduce in the TME of these fish as demonstrated by decreased transcript levels of *lck*, *cd4-1*, and *cd8*α (Dee et al., 2016). Further analysis of tumor-infiltrating Cd4-1 + cells showed that these cells expressed higher levels of *gata3* and *il-4/13a* but not *il-4/13b*, indicating the presence of a specific subset of Th2 cells in the TME of melanoma, which are absent in normal mucosal environments such as the gills (Dee et al., 2016). Gómez-Abenza et al. sought to understand the TME using the zebrafish model of melanoma in the context of chronic inflammation (Gómez-Abenza et al., 2019). Through the use of a zebrafish line deficient of serine peptidase inhibitor kunitz type 1 (SPINT1), they showed that SPINT1 levels positively correlated with rates of macrophage infiltration into the TME, indicating its regulation of the crosstalk between tumor cells and the immune system. These findings demonstrate that zebrafish is suitable to investigate the interplays among cancer cells, innate, and adaptive immune system.

### Zebrafish Xenografts to Screen for Immunomodulators and Test Cell-Based Immunotherapies

Similar to zebrafish genetic models of cancer, researchers also utilize differential labeling of immune and tumor cells in zebrafish xenograft models of cancer to visualize immune-tumor cell interactions *in vivo*. In addition, the small size and a large number of xenografted embryos enable rapid screens and evaluation of compounds that modulate tumor-immune cell interactions. In particular, the simultaneous introduction of both human cancer and immune cells from the same patient enables the evaluation and selection of optimal cell-based immunotherapy, such as CAR-T cells. For instance, Yan et al. have recently generated a *Casper*; *prkdc-/-*; *il2rga-/-* mutant fish that can be reared at 37°C to better replicate human conditions and support a wide variety of cancer cell engraftments, including patient-derived human cells (Yan et al., 2019). Their work demonstrated the feasibility of this model to assess the efficacy of CAR-T cells in eradicating cancer cells. Pascoal et al. showed that zebrafish xenografts can be used to screen optimal CAR-T cell therapies (Pascoal et al., 2020). Researchers have also utilized zebrafish xenografts to visualize the killing of dormant metastatic cancer cells by CAR-T cells *in vivo* at the single-cell resolution (He et al., 2020). Al-Samadi et al. (2017) demonstrated that extracellular vesicles isolated from human tongue carcinoma cells can trigger the diminishment of *il-13* mRNA in zebrafish, which is an anti-inflammatory cytokine. These studies provide evidence to support the feasibility of zebrafish xenografts in immunomodulator screening and cell-based immunotherapy testing, demonstrating their potential to bridge *in vitro* studies and those conducted in mammals like mice (He et al., 2020; Pascoal et al., 2020).

## Future Perspectives

In summary, the zebrafish has served as one of the key model organisms in recent decades to further our understanding of cancer etiologies and immune system development in vertebrates (Trede et al., 2004; Hason and Bartůněk, 2019; Rossa and D’Silva, 2019). Comparative studies of hematopoiesis demonstrated similarities in the genetic and cellular components of the immune system with only a few differences in the timing and location of the development. Furthermore, these studies revealed surprisingly high conservation in the adaptive immune system between zebrafish and humans. Distinct γδ T cells, cytotoxic CD8 + T cells, CD4 + T cells, and their subtypes are present in both species. In addition, zebrafish and humans share similar differentiation patterns and functions of B lymphocytes, with some differences in the classes and abundance of immunoglobulins as well as the absence of pre-B cells in zebrafish. The overarching similarities between the zebrafish and humans have encouraged the development of a myriad of technologies and tools available for tumor immunity research.

The zebrafish possesses unique advantages for tumor immunity research. The optical clarity of zebrafish together with their small size and low cost makes imaging studies of the TME and screening of immunomodulators feasible. Moreover, the *ex-utero* development of the zebrafish embryos allows easy and non-invasive access to image the TME, especially tumor-immune cell interactions, at the early stages of tumor development. An in-depth investigation of how the immune system evolves from the anti-tumor to pro-tumor stage holds the key to unlocking the mechanisms of immune evasion. For zebrafish embryos or juveniles under 3 weeks of age, they cannot mount an adaptive immune response (Rossa and D’Silva, 2019). This temporal lag in the development of innate and adaptive immunity in zebrafish is useful to study the differences in interactions between the innate and adaptive immune systems with tumor cells (Rossa and D’Silva, 2019). Moreover, the development of immunocompromised zebrafish mutants in which specific adaptive immune components are removed facilitates elegant probing of these individual component’s contribution to tumor development.

Like any other model system, the zebrafish also possesses some limitations. Gene duplication within the zebrafish genome, which can lead to redundancy in gene function, creates difficulties in gene knockout studies (Postlethwait et al., 2000; Taylor et al., 2003; Howe et al., 2013). As mentioned above, some differences also exist during immune cell development between zebrafish and humans. Importantly, preliminary work using zebrafish and other teleosts have provided fresh mechanistic insights into the regulation of the TME (Dee et al., 2016; Bjørgen et al., 2019; Gómez-Abenza et al., 2019), along with demonstrations of the feasibility of zebrafish xenografts for screening personalized treatments (Al-Samadi et al., 2017; Yan et al., 2019; Pascoal et al., 2020). With its technological advances, established research tools, and high levels of conservation, the zebrafish has joined murine models and human genetic studies for tumor immunology research to uncover novel immunotherapeutic strategies for improved cancer treatment.

## Author Contributions

KM, GK, GM, XQ, and HF wrote and edited the manuscript. All authors contributed to the article and approved the submitted version.

## Conflict of Interest

The authors declare that the research was conducted in the absence of any commercial or financial relationships that could be construed as a potential conflict of interest.
